# Obicetrapib exhibits favorable physiochemical and pharmacokinetic properties compared to previous cholesteryl ester transfer protein inhibitors: An integrated summary of results from non‐human primate studies and clinical trials

**DOI:** 10.1002/prp2.70010

**Published:** 2024-10-18

**Authors:** Stephen J. Nicholls, Adam J. Nelson, John J. P. Kastelein, Marc Ditmarsch, Andrew Hsieh, Judith Johnson, Danielle Curcio, Douglas Kling, Carol F. Kirkpatrick, Michael H. Davidson

**Affiliations:** ^1^ Victorian Heart Institute Monash University Melbourne Victoria Australia; ^2^ NewAmsterdam Pharma B.V Naarden The Netherlands; ^3^ Midwest Biomedical Research Addison Illinois USA

**Keywords:** accumulation, anacetrapib, cholesteryl ester transfer protein, elimination, obicetrapib, pharmacokinetics, toxicokinetic

## Abstract

Anacetrapib, a cholesteryl ester transfer protein (CETP) inhibitor previously under development, exhibited an usually extended terminal half‐life and large food effect and accumulated in adipose tissue. Other CETP inhibitors have not shown such effects. Obicetrapib, a potent selective CETP inhibitor, is undergoing Phase III clinical development. Dedicated assessments were conducted in pre‐clinical and Phase I and II clinical studies of obicetrapib to examine the pharmacokinetic issues observed with anacetrapib. After 9 months of dosing up to 50 mg/kg/day in cynomolgus monkeys, obicetrapib was completely eliminated from systemic circulation and not detected in adipose tissue after a 13‐week recovery period. In healthy humans receiving 1–25 mg of obicetrapib, the mean terminal half‐life of obicetrapib was 148, 131, and 121 h at 5, 10, and 25 mg, respectively, and food increased plasma levels by ~1.6‐fold with a 10 mg dose. At the end of treatment in Phase II trials, mean plasma levels of obicetrapib ranged from 194.5 ng/mL with 2.5 mg to 506.3 ng/mL with 10 mg. Plasma levels of obicetrapib decreased by 92.2% and 98.5% at four and 15 weeks post‐treatment, respectively. Obicetrapib shows no clinically relevant accumulation, is minimally affected by food, and has a mean terminal half‐life of 131 h for the 10 mg dose. These data support once daily, chronic dosing of obicetrapib in Phase III trials for dyslipidemia management.

## Graphical abstract

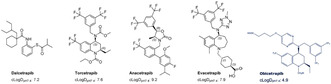

**Abbreviations**
Apo: apolipoprotein
ASCVD: atherosclerotic cardiovascular disease
AUC: area under the curve
BMI: body mass index
BROADWAY: Randomized Study to Evaluate the Effect of Obicetrapib on Top of Maximum Tolerated Lipid‐Modifying Therapies
BROOKLYN: Evaluate the Effect of Obicetrapib in Patients with Heterozygous Familial Hypercholesterolemia on Top of Maximum Tolerated Lipid‐Modifying Therapies
CETP: cholesteryl ester transfer protein
cLogD: calculated logarithm of the distribution coefficient
*C*_max_
: maximum plasma concentration
CSR: Clinical Study Report
DA VINCI: EU‐Wide Cross‐Sectional Observational Study of Lipid‐Modifying Therapy Use in Secondary and Primary Care
DEFINE: Determining the Efficacy and Tolerability of CETP Inhibition with Anacetrapib
GOULD: Getting to an Improved Understanding of Low‐Density Lipoprotein Cholesterol and Dyslipidemia Management
HDL: high‐density lipoprotein
HDL‐C: high‐density lipoprotein cholesterol
LDL: low‐density lipoprotein
LDL‐C: low‐density lipoprotein cholesterol
LLOQ: lower limit of quantification
mITT: modified Intent‐to‐Treat
PK: pharmacokinetic
PREVAIL: Cardiovascular Outcome Study to Evaluate the Effect of Obicetrapib in Patients with Cardiovascular Disease
PSA: polar surface area
R_ac_
: accumulation ratio
REVEAL: Randomized Evaluation of the Effects of Anacetrapib through Lipid Modification
ROSE: Randomized Study of Obicetrapib as an Adjunct to Statin Therapy
ROSE2: Study to Evaluate the Effect of Obicetrapib in Combination with Ezetimibe as an Adjunct to High‐Intensity Statin Therapy
*t*_1/2_
: terminal half‐life
TA‐8995‐203: A Dose‐Finding Study in Japanese Patients to Evaluate the Effect of Obicetrapib as an Adjunct to Stable Statin Therapy
TANDEM: Study of Obicetrapib and Ezetimibe Fixed Dose Combination on Top of Maximum Tolerated Lipid‐Modifying Therapies
*t*_max_, time to maximum plasma concentration: time to maximum plasma concentration
TULIP: TA‐8995: Its Use in Patients with Mild Dyslipidaemia

## 1 INTRODUCTION

Cholesteryl ester transfer protein (CETP) is a hydrophobic glycoprotein synthesized primarily in the liver, adipose tissue, and spleen.^1^, ^2^ CETP mediates the transfer of cholesteryl esters from high‐density lipoprotein (HDL) particles to apolipoprotein (Apo) B‐containing particles (i.e., low‐density lipoprotein [LDL] and very‐low‐density lipoprotein) and triglycerides from ApoB‐containing particles to HDL. The results of randomized controlled trials of early CETP inhibitors demonstrated that inhibiting CETP significantly increased HDL cholesterol (HDL‐C) levels (torcetrapib, dalcetrapib, evacetrapib, and anacetrapib) and significantly reduced ApoB‐containing lipoprotein levels (torcetrapib, evacetrapib, and anacetrapib), compared to placebo.^3^, ^4^, ^5^, ^6^ Obicetrapib is a next generation, highly selective CETP inhibitor that was developed as a tetrahydroquinoline derivative with a pyrimidine and an ethoxycarbonyl structure with two chiral centers to improve the physical and biopharmaceutical properties compared to previous CETP inhibitors.^7^, ^8^ Obicetrapib potently inhibits CETP activity,^9^ resulting in significant reductions in levels of ApoB and ApoB‐containing lipoprotein cholesterol [LDL‐C, non‐HDL‐C, and lipoprotein(a)], as well as significant increases in HDL‐C and ApoA‐I. Thus far, obicetrapib has had a safety and tolerability profile comparable to placebo when used as monotherapy or on top of statins and/or in combination with ezetimibe in Phase I and Phase II trials.^9^, ^10^, ^11^, ^12^, ^13^

The results of the Randomized Evaluation of the Effects of Anacetrapib through Lipid Modification (REVEAL) trial demonstrated a significant 9% reduced risk of major adverse coronary events when anacetrapib was added to high‐intensity statin therapy, compared to placebo.^6^ This finding supports the view that CETP inhibition reduced coronary risk by lowering ApoB‐containing lipoproteins, not by increasing HDL‐C.^14^, ^15^ However, the sponsor did not apply for regulatory approval for anacetrapib after a review of its clinical profile,^16^ a decision most likely related to its extended terminal half‐life (*t*_1/2_) with chronic dosing and accumulation in adipose tissue.^17^, ^18^

Although early‐phase clinical trials of anacetrapib demonstrated a relatively short half‐life (up to 80 h after 14 days of dosing),^19^ pharmacokinetic (PK) analyses in subsequent trials of longer treatment duration demonstrated a longer elimination half‐life.^7^ In a Phase IIb trial, plasma levels of anacetrapib were measured 8 weeks after the last treatment dose and were 18–26% of plasma levels observed during the treatment phase, which suggested a *t*_1/2_ of 3–4 weeks, assuming mono‐exponential decay.^20^ In the Determining the Efficacy and Tolerability of CETP Inhibition with Anacetrapib (DEFINE) trial, which had a duration of 76 weeks, plasma levels of anacetrapib were 7–8% of steady‐state on‐treatment plasma levels up to 4 years after the last treatment dose.^7^, ^21^ Small et al. suggested that the longer duration of DEFINE improved the ability to identify the true terminal phase of anacetrapib.^22^ The results of studies that examined subcutaneous adipose biopsy measurements demonstrated that anacetrapib accumulated in adipose tissue at much higher concentrations and for a prolonged period compared to plasma levels due to a slower elimination rate from adipose tissue.^7^, ^17^, ^22^

The long *t*_1/2_ and accumulation in adipose tissue seem to be unique to anacetrapib^18^ and appear to be related to its physiochemical properties. Anacetrapib has the highest lipophilicity and lowest polar surface area (PSA) of CETP inhibitors,^23^ which contribute to a higher potential for its distribution into adipose tissue.^22^ These physicochemical properties also explain the increased plasma levels of anacetrapib that were observed in a fed versus fasted state, especially after a high‐fat meal.^19^, ^22^, ^24^ In comparison, PK assessments of the other CETP inhibitors demonstrated a *t*_1/2_ of 211 h for torcetrapib in a single ascending dose study,^25^ 30 h for dalcetrapib after once daily doses between 300 and 1200 mg in short‐term investigations,^26^ and 40 h for evacetrapib after once daily doses between 10 and 600 mg for up to 15 days,^27^ and no accumulation in adipose tissue.^7^, ^22^ Torcetrapib is the only CETP inhibitor that exhibited a larger food effect than anacetrapib, with a 20‐ to 30‐fold increase in torcetrapib plasma levels^28^ compared to ~2‐ to 8‐fold increase in anacetrapib levels^24^ in a fed versus fasted state. Dalcetrapib and evacetrapib exhibited smaller food effects of ~1.3‐ to 1.5‐fold increase in plasma levels in a fed versus fasted state.^29^, ^30^

Based on the accumulation issues identified with anacetrapib, studies with cynomolgus monkeys and Phase I and II trials of obicetrapib in humans were used to examine its definitive elimination. The purpose of this article is to summarize the results of these studies, which have supported the progression of obicetrapib into Phase III clinical development.^8^

## 2 METHODS

### 2.1 Study in cynomolgus monkeys

CETP is present and active in non‐human primates, rabbits, and hamsters, but is absent in most other animal species, including mice, rats, and dogs.^2^, ^31^, ^32^ A non‐human primate study was conducted using cynomolgus monkeys (*Macaca fascicularis*) to evaluate the toxicity of obicetrapib (TA‐8995), following twice daily (b.i.d.), six h apart, oral (gavage) administration to the monkey for 39 weeks (Clinical Study Report [CSR], NewAmsterdam Pharma). The monkey was selected as the species for this study because the CETP structure in monkeys is very similar to the structure of human CETP.^33^ The cynomolgus monkeys were obtained from Noveprim Ltd, Mauritius. The monkeys were approximately 26–30 months old when obtained for the study and 28–32 months old at the start of dosing. On arrival at the study site, the weight range of the monkeys was 2.4–3.3 kg. Individual body weights were recorded once weekly (including Day 1 before the start of dosing) from time of allocation and on the day of necropsy. At the start of dosing, the weight range of the monkeys was 2.4–3.3 kg and 2.5–3.9 kg for the females and males, respectively. The weight range of the monkeys at the end of the dosing period (Week 39) was 3.0–4.8 kg and 3.0–5.2 kg for females and males, respectively.

Group mean body weights for treated monkeys were comparable with the control monkeys. The weight range of the recovery monkeys (*n* = 8) at Week 14 of the recovery period, prior to necropsy, was 3.5–5.2 kg and 3.7–4.9 kg for the females and males, respectively. During the recovery period, mean body weights for the monkeys treated at 50 mg/kg/day (25 mg/kg/b.i.d.) during dosing were comparable with the control monkeys (CSR, NewAmsterdam Pharma).

The animals were housed in pens that conform to the “Code of practice for the housing and care of animals used in scientific procedures” in rooms exclusive to the study. The rooms were air‐conditioned to provide 15–20 air changes/hour, and the temperature and relative humidity ranges were maintained at 21–25°C and 40–70%, respectively. Fluorescent lighting was controlled automatically to give a cycle of 12 h light and 12 h dark, with the exception of when experimental procedures dictated otherwise. A number of strategies were used for environmental enrichment for the monkeys, such as the provision of toys (e.g., balls, inert nylon chews), swings, and foraging materials. The monkeys were offered ad libitum water, 100 g of SQC Mazuri Primate Diet (Special Diets Services Ltd., Witham, UK), and a 25 g Bonio biscuit (Spillers) each day. When available, the diet was supplemented twice daily with fresh fruit, fresh vegetables, forage mix, peanuts, raisins, or sunflower seeds. The study was conducted in accordance with the requirements of the Animals (Scientific Procedures) Act 1986 and a local ethical review was maintained (CSR, NewAmsterdam Pharma).

The monkeys (*n* = 40; 20 of each sex) were allocated into four groups (based on existing social groupings) to receive treatment for 39 weeks: a control group (no treatment), a low‐dose group (10 mg/kg/day [5 mg/kg/b.i.d.] of obicetrapib), an intermediate‐dose group (20 mg/kg/day [10 mg/kg/b.i.d.] of obicetrapib), and a high‐dose group (50 mg/kg/day [25 mg/kg/b.i.d.] of obicetrapib). Four monkeys (two of each sex) from the 50 mg/kg/day dose group and four (two of each sex) from the control group were followed for an extended treatment‐free period of 13 weeks (recovery period). Obicetrapib was dosed as a suspension in 0.5% sodium carboxylmethyl cellulose solution and 0.1% polyoxyethylene hydrogenated castor oil. Toxicokinetic parameters were examined after 39 weeks of dosing. Blood samples for toxicokinetic evaluation were taken from all monkeys in the study on Day 1 and Day 273 (Week 39) pre‐dose and at 0.5, 1, 2, 3, 4, 6, 7.5, 9, 10, 12, and 24 h after dosing. Blood samples for toxicokinetic assessments were taken from the recovery animals on multiple days during the treatment‐free period at the same time intervals as the 24‐hour samples in Week 39. Delayed onset toxicity and/or reversibility of toxicity were examined during the 13‐week treatment‐free period. The assessment of toxicity was based on mortality, clinical and post‐dose observations, body weight, rectal temperature, ophthalmoscopy, electrocardiography, and clinical and anatomic pathology evaluations. Samples of liver (5 g nominal) and perirenal white fat (up to 1 g) were collected from the recovery animals at necropsy after the 13‐week treatment‐free period (CSR, NewAmsterdam Pharma).

### 2.2 Clinical trials

The PK parameters and the effect of food on the bioavailability of obicetrapib in humans have been examined in both Phase I^9^ (TA‐8995‐09 CSR, NewAmsterdam Pharma) and Phase II trials.^10^, ^11^, ^12^, ^13^ The trials were performed in accordance with the Declaration of Helsinki, Good Clinical Practice Guidelines, and applicable regulatory requirements. Approval was obtained from the relevant ethical review boards for each site. All volunteers provided written informed consent prior to participation. PK parameters were determined using WinNonlin software, version 4.1 or higher (Pharsight/Certara Corporation, USA). Concentrations of obicetrapib were summarized for each treatment group using descriptive statistics.

#### 2.2.1 Phase I trials assessing obicetrapib PK parameters

Two Phase I trials were conducted to examine the tolerability, PK parameters, and pharmacodynamics of obicetrapib in healthy participants. The first trial (NCT01878474) was a double‐blind, randomized single dose trial that included 12 groups of healthy participants who received obicetrapib or placebo while under fasting conditions: (groups 1–6) White male participants (aged 18–55 years) who received single ascending doses of 5, 10, 25, 50 (participants in the 50 mg dose group received the same dose after a high‐fat meal 4 weeks after the fasting dose), 100, and 150 mg obicetrapib; (group 7) White males (aged >65 years) who received a single dose of 25 mg obicetrapib; (group 8) White females (aged 18–55 years) who received a single dose of 25 mg obicetrapib; and groups (9–12) Japanese males (aged 18–55 years) who received single ascending doses of 25, 50, 100, and 150 mg obicetrapib. Participants in each group/dose level were allocated to treatment in a ratio of 6:2 of obicetrapib to placebo. Blood samples for PK assessments were collected prior to each dose and at intervals up to 336 h post‐dose. Urine was collected for PK assessment pre‐dose and at intervals up to 72 h postdose from selected dose groups.^9^

The second trial (NCT01879020) was a double‐blind, randomized multiple ascending dose trial that included five groups of healthy White male participants (aged 18–55 years) who received a daily dose of obicetrapib or placebo after a standard breakfast: (group 1) single oral dose of 5 mg on day one, and then repeated daily doses on days eight to 35; and groups (2–5) single oral dose of 1, 2.5, 10, or 25 mg obicetrapib on day one, and then repeated daily doses on days eight to 28. The participants were allocated to the treatment groups in a ratio of 10:2 of obicetrapib to placebo. Blood samples for PK assessments were collected prior to each dose and at intervals throughout the trial until 336 h after the last dose. Urine was collected for PK assessments from pre‐dose and at intervals up to 72 h after the first and last doses.^9^

#### 2.2.2 Phase I trial assessing food effect on obicetrapib bioavailability (TA‐8995‐09)

This was an open‐label, randomized, two‐sequence, two‐period, two‐treatment crossover study conducted to evaluate the effect of food on the bioavailability of 10 mg obicetrapib. Thirty healthy adult males and females were randomized to treatment and data were available for 23 participants. In Treatment Period I, participants received a single 10 mg dose of obicetrapib after an overnight fast. In Treatment Period II, participants received a single 10 mg dose of obicetrapib at 30 min following the start of a standardized high‐fat, high‐calorie breakfast that was preceded by an overnight fast. Blood samples were collected at pre‐dose and at intervals over 336 h after dosing in each treatment period. The interval between doses was 49 days. The half‐life of obicetrapib was expected to be less than 6 days; therefore, it was anticipated that 49 days would be well beyond five half‐lives and an adequate washout period (TA‐8995‐09 CSR, NewAmsterdam Pharma).

#### 2.2.3 Phase II trials

The Phase II trials of obicetrapib described herein include the TA‐8995: Its Use in Patients with Mild Dyslipidaemia (TULIP)^10^ study, the Randomized Study of Obicetrapib as an Adjunct to Statin Therapy (ROSE),^11^ the Study to Evaluate the Effect of Obicetrapib in Combination with Ezetimibe as an Adjunct to High‐Intensity Statin Therapy (ROSE2),^12^ and A Dose‐Finding Study in Japanese Patients to Evaluate the Effect of Obicetrapib as an Adjunct to Stable Statin Therapy (TA‐8995‐203).^13^

#### 2.2.4 TULIP

TULIP (NCT01970215) was a placebo‐controlled, double‐blind, randomized parallel‐group trial conducted to evaluate the effects of varying doses of obicetrapib as monotherapy and in combination with moderate‐intensity statin therapy on LDL‐C and HDL‐C levels.^10^

Additionally, the plasma concentrations of obicetrapib were evaluated during and after the treatment period. Eligible participants had mild dyslipidemia defined as LDL‐C levels between 96–174 mg/dL, HDL‐C levels between 31 and 70 mg/dL, and triglyceride levels <400 mg/dL after a washout for those on lipid‐modifying therapies. Three hundred and sixty‐four participants (mean age: 64.7 years; 81.6% male; 98.1% White; mean body mass index [BMI]: 26.0 kg/m^2^) were enrolled at 17 clinical research sites in the Netherlands and Denmark and randomized in a 1:1 ratio to one of nine treatment groups: (1) placebo; (2) 1 mg obicetrapib; (3) 2.5 mg obicetrapib; (4) 5 mg obicetrapib; (5) 10 mg obicetrapib; (6) 10 mg atorvastatin; (7) 10 mg obicetrapib plus 20 mg atorvastatin; (8) 10 mg rosuvastatin; or (9) 10 mg obicetrapib plus 10 mg rosuvastatin. The participants were instructed to take their assigned treatment orally with food once daily for the 12‐week treatment period. After randomization, participants returned to the clinical research site for PK assessments at Weeks 8 and 12 (end of treatment). Participants also completed follow‐up visits two and eight weeks after the end of treatment (Weeks 14 and 20) for final safety and PK assessments. Of the 364 participants randomized, 337 (92.6%) completed the trial and were included in the primary analysis. The PK population (*n* = 242) included all randomized participants who received at least one dose of obicetrapib and had at least one valid plasma drug concentration data assessment; 227 (93.8%) of the participants in the PK population were included in the 8‐week post‐end of treatment PK analysis (TA‐8995‐03 CSR, NewAmsterdam Pharma).

#### 2.2.5 ROSE

ROSE (NCT04753606) was a placebo‐controlled, double‐blind, randomized trial conducted to evaluate the efficacy of obicetrapib as an adjunct to high‐intensity statin therapy for reducing LDL‐C and ApoB levels.^11^ A secondary endpoint of the trial included assessing plasma concentrations of obicetrapib during and after the end of treatment. Eligible participants had an LDL‐C level >70 mg/dL and triglyceride levels <400 mg/dL and were receiving high‐intensity statin therapy (either 40 or 80 mg atorvastatin or 20 or 40 mg rosuvastatin) for at least 8 weeks prior to screening, which they continued throughout the treatment period. One hundred twenty participants (mean age: 61.8 years; 55.8% male; 76.7% White; mean BMI: 31.1 kg/m^2^) were enrolled at 17 clinical research sites in the United States and randomized in a 1:1:1 ratio to one of three treatment groups: (1) placebo; (2) 5 mg obicetrapib; or (3) 10 mg obicetrapib. The participants were instructed to take their assigned treatment orally with food once daily at approximately the same time each morning for the 8‐week treatment period. After randomization, participants returned to the clinical research site during Weeks 4 and 8 (end of treatment) for PK assessments. Additionally, participants completed follow‐up visits at four, eight, and 15 weeks after the end of treatment (Weeks 12, 16, and 23) for PK assessments. Of the 120 participants randomized in ROSE, 119 (99.2%) completed treatment; all 120 participants randomized were included in the modified Intent‐to‐Treat (mITT) analysis. The PK population (*n* = 80) included all randomized participants in the mITT population assigned to the two obicetrapib dose groups who had sufficient blood samples collected for valid estimation of PK parameters. Seventy‐seven (96.3%), 79 (98.8%), and 79 (98.8%) participants in the PK population for ROSE were included in the 4‐, 8‐, and 15‐week post‐end of treatment PK analyses, respectively (TA‐8995‐201 CSR, NewAmsterdam Pharma).

#### 2.2.6 ROSE2

ROSE2 (NCT05266586) was a placebo‐controlled, double‐blind, randomized trial conducted to evaluate the efficacy, safety, and tolerability of obicetrapib or obicetrapib plus ezetimibe as an adjunct to high‐intensity statin therapy.^12^ An exploratory endpoint of the trial included assessing the plasma concentrations of obicetrapib during treatment and 4 weeks after the end of treatment. Eligible participants had a fasting LDL‐C level >70 mg/dL and triglyceride levels <400 mg/dL and were receiving a stable dose of a high‐intensity statin (40 or 80 mg atorvastatin or 20 or 40 mg rosuvastatin) for at least 8 weeks prior to screening and throughout the treatment period. One hundred nineteen participants (mean age: 62.6 years; 63.9% male; 84.5% White; mean BMI: 30.9 kg/m^2^) were enrolled at 18 clinical research sites in the United States and randomized in a 1:1:1 ratio to one of three treatment groups: (1) placebo; (2) obicetrapib monotherapy (one 10 mg obicetrapib tablet plus one placebo capsule for ezetimibe); or (3) combination therapy (one 10 mg obicetrapib tablet plus one 10 mg ezetimibe capsule). The participants were instructed to take their assigned treatment orally with water once daily at approximately the same time each morning for the 12‐week treatment period. After randomization, participants returned to the clinical research site during Weeks 4 and 12 (end of treatment) for safety and PK assessments. Participants also completed a follow‐up visit 4 weeks after the end of treatment (Week 16) for final safety and PK assessments. Of the 119 participants randomized, 97 (81.5%) were included in an on‐treatment analysis, which excluded participants with plasma concentrations of obicetrapib <100 ng/mL at either or both Week 4 and Week 12.^12^ The on‐treatment PK population (*n* = 57) included all randomized participants in the mITT population assigned to the two obicetrapib dose groups who had sufficient blood samples collected for valid estimation of PK parameters and excluded participants with plasma concentrations of obicetrapib <100 ng/mL at either or both Week 4 and Week 12. Fifty‐six (98.2%) participants in the on‐treatment PK population were included in the 4‐week post‐end of treatment PK analysis (TA‐8995‐202 CSR, NewAmsterdam Pharma).

#### 2.2.7 TA‐8995‐203

This Phase II trial (NCT05421078) was designed to evaluate the efficacy, safety, and tolerability of obicetrapib as an adjunct to stable statin therapy in Japanese participants with LDL‐C >70 mg/dL, or non‐HDL‐C >100 mg/dL, and triglyceride levels <400 mg/dL.^13^ Eligible participants were receiving either atorvastatin 10 or 20 mg or rosuvastatin 5 or 10 mg for at least 8 weeks prior to screening and continued statin therapy throughout the treatment period. One hundred two participants (mean age: 64.8 years; 71.6% male; mean BMI: 25.6 kg/m^2^) were enrolled at nine clinical research sites in Japan and randomized to one of four treatment groups in a 1:1:1:1 ratio: 1) placebo; 2) 2.5 mg obicetrapib; 3) 5 mg obicetrapib; or 4) 10 mg obicetrapib.

The participants were instructed to take their assigned treatment orally once daily at approximately the same time each morning for the 8‐week treatment period. After randomization, participants returned to the clinical research site during Weeks 2, 4, and 8 (end of treatment) for PK assessments. Participants also completed a follow‐up visit 4 weeks after the end of treatment (Week 12) for a final PK assessment. All of the participants who were randomized completed the treatment and were included in the primary analysis. The PK population included all randomized participants in the mITT population assigned to the three obicetrapib dose groups who had sufficient blood samples collected for valid estimation of PK parameters; 76 (100%) participants in the PK population were included in the 4‐week post‐end of treatment PK analysis (TA‐8995‐203 CSR, NewAmsterdam Pharma).

Key protein targets and ligands in this article are hyperlinked to corresponding entries in http://www.guidetopharmacology.org, the common portal for data from the IUPHAR/BPS Guide to PHARMACOLOGY,^34^ and are permanently archived in the Concise Guide to PHARMACOLOGY 2023/24.^35^, ^36^

## 3 RESULTS

### 3.1 Study in cynomolgus monkeys

**FIGURE 1:**
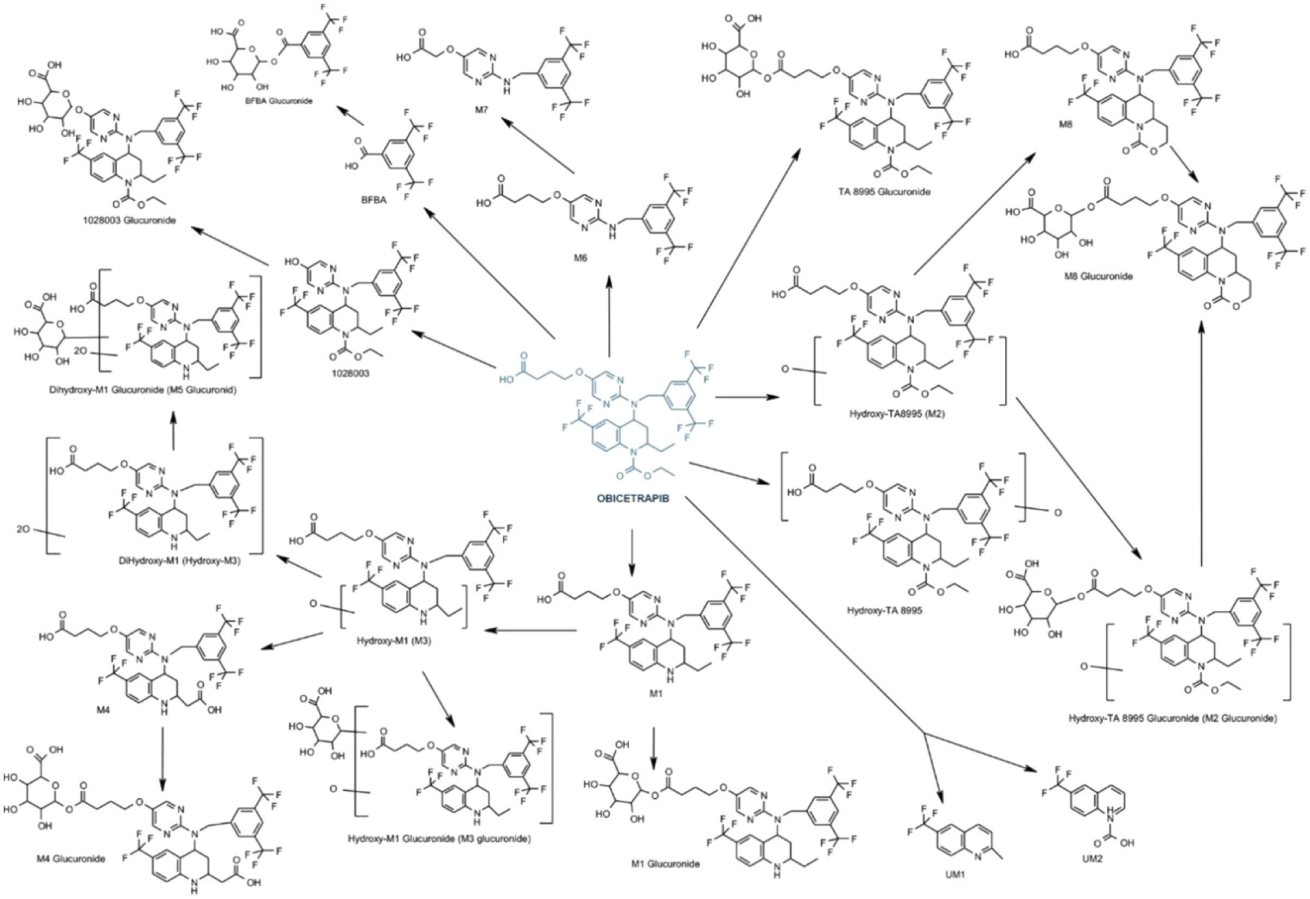
Metabolism of obicetrapib (TA‐8995) in the cynomolgus monkey (*n*, 3). M, metabolite; UM, urinary metabolite.

Figure 1 illustrates the metabolism of obicetrapib (TA‐8995) in the cynomolgus monkey. A study was conducted to examine the disposition of ^14^C‐obicetrapib following oral administration in the cynomolgus monkey (*n* = 3 males) at a dose level of 20 mg/kg. One of the objectives of the study was to determine the relative proportions of ^14^C‐obicetrapib and its radiolabeled metabolites (M1) in plasma using liquid chromatography mass spectrometry. The monkeys each received a single oral dose of ^14^C‐obicetrapib by gavage at a target dose level of 20 mg/kg and a nominal dose volume of 5 mL/kg. Blood samples were collected at specified times from 0 to 336 h. After the final blood collection, radioactivity was measured in the blood and plasma by liquid scintillation counting. The profile of M1 in selected samples of plasma was determined by high‐performance liquid chromatography (HPLC) (following extraction of the matrix, where relevant) and selected M1 were identified by liquid chromatography mass spectrometry. In plasma, unchanged obicetrapib and a M1 were the principal radioactive components and represented at least 66% of the chromatographic radioactivity in any single profile.

Additional M1 were identified in urine and feces samples, indicating that the metabolism of obicetrapib was extensive in the cynomolgus monkey (Figure 1) (CSR, NewAmsterdam Pharma).

Table 1 summarizes the toxicokinetic parameter results for Day 1 compared to the end‐of‐treatment (Day 273) for the toxicokinetic study. At the end of treatment, the mean maximum plasma concentration (*C*_max_) of obicetrapib was achieved within 9 h (3 h after the second dose) in all of the monkeys. In the male cynomolgus monkeys, the mean *C*_max_ was 3800, 6410, and 9210 ng/mL for the 10, 20, and 50 mg/kg/day doses, respectively. In the female monkeys, the mean *C*_max_ was 3390, 5160, and 8480 ng/mL for the 10, 20, and 50 mg/kg/day doses, respectively. Mean accumulation ratios (R_ac_; Week 39/Day 1) for obicetrapib ranged from 0.8 to 1.4 for *C*_max_, and 1.0 to 1.4 for the area under the plasma concentration‐versus‐time curve calculated from 0 to 24 h [AUC_(0–24 h)_]. An R_ac_ of 1.2 ≤ R_ac_ <2 indicates weak accumulation.^37^ Thus, the R_ac_ determined in this study indicate minimal accumulation at the dosages used over 39 weeks. On Day 1, the ratios of male/female for *C*_max_ and AUC_(0–24)_ were between 0.8 and 1.0. In Week 39, the ratios of male/female for *C*_max_ and AUC_(0–24)_ were between 1.0 to 1.2. Based on visual inspection, these results suggest that the exposure to obicetrapib was similar in males and females. The sample size was not large enough to complete meaningful subgroup statistical comparisons.

**TABLE 1:** Summary of toxicokinetic parameter results^a^ for obicetrapib (TA‐8995) from a study in non‐human primates (CSR, Data on file, NewAmsterdam Pharma).

	Study Groups and Treatment Dose					
	10 mg/kg/d		20 mg/kg/d		50 mg/kg/d	
	Males	Females	Males	Females	Males	Females
Parameter	(*n* = 4)	(*n* = 4)	(*n* = 4)	(*n* = 4)	(*n* = 6)	(*n* = 6)
Day 1						
*C*_max_ (ng/mL)	2800	3300	4740	4980	9600	11 600
	(412)	(289)	(1080)	(1100)	(2330)	(3110)
*t*_max_ (h)	5.0 (2.9)	5.9 (3.3)	2.5 (0.6)	4.8 (3.2)	7.6 (2.0)	6.2 (2.1)
*t*_½_ (h)	6.8 (1.0)	7.2 (0.8)	6.9 (2.4)	5.8 (1.1)	6.8 (1.7)	6.8 (1.7)
AUC_(0–24)_ (ng·h/mL)	29 300	29 800	46 600	47 800	118 000	118 000
	(8290)	(5040)	(8370)	(11 300)	(28 900)	(39 500)
Day 273 (Week 39)						
*C*_max_ (ng/mL)	3800	3390	6410	5160	9210	8480
	(604)	(843)	(1310)	(1320)	(1410)	(1270)
*t*_max_ (h)	2.0 (NC)	4.8 (3.2)	2.3 (0.5)	4.8 (3.2)	8.5 (0.8)	6.6 (3.2)
*t*_½_ (h)	11.0 (2.3)	12.1 (4.3)	8.1 (2.5)	8.8 (2.5)	6.4 (1.5)	7.4 (1.5)^b^
AUC_(0–24)_ (ngh/mL)	40 300	41 300	65 200	54 000	111 000	103 000
	(6710)	(10 800)	(9110)	(19 800)	(24 000)	(19 500)
R_ac_ *C*_max_ ^c^	1.4 (0.1)	1.0 (0.2)	1.4 (0.3)	1.1 (0.2)	1.0 (0.2)	0.8 (0.1)
R_ac_ AUC_(0–24 h)_ ^c^	1.4 (0.3)	1.4 (0.2)	1.4 (0.2)	1.1 (0.2)	1.0 (0.1)	0.9 (0.2)

Abbreviations: AUC_(0–24)_, area under the plasma concentration‐versus‐time curve calculated from 0 to 24 h; *C*_max_, maximum plasma concentration; NC, not calculated; R_ac_, accumulation ratio; *t*_½_, elimination half‐life; *t*_max_, time to maximum plasma concentration.

^a^ Values are reported as the mean (standard deviation).

^b^ Does not include data from one outlier.

^c^ Accumulation ratios (R_ac_) for AUC_(0–24 h)_ and *C*_max_ were calculated by dividing the value in Week 39 by the corresponding value on Day 1.

The mean *t*_1/2_ in the monkeys ranged from 5.8 to 7.2 h on Day 1 and 6.4 to 12.1 on Day 273 (Table 1). Apparent *t*_1/2_ for the individual monkeys ranged between 4.8 and 10.5 h on Day 1 and between 5.3 and 18.3 h in Week 39, with one exception. One female in the 50 mg/kg/day group had detectable concentrations of obicetrapib at the end of the recovery period (13 weeks after the last dose) and had a *t*_½_ of 516 h. This resulted in a mean *t*_1/2_ value of 109 h for the females in the high dose group. When this value was excluded from the calculations, the mean *t*_1/2_ was reduced to 7.4 h, which is consistent with the lower dose groups and also the males in the high dose group. Therefore, the results for the mean *t*_1/2_ for the female monkeys in the 50 mg/kg/day group excluded the value from the outlier.

At the end of the 13‐week treatment‐free period, the plasma levels of the four recovery monkeys (two of each sex) from the 50 mg/kg/day dose group demonstrated that elimination of obicetrapib was complete in both males and one female; a minimal amount of obicetrapib (0.25% of the observed maximum concentration) was detected in the other female. Furthermore, the results of the adipose tissue analysis indicated that, in all four of these recovery monkeys, obicetrapib was below the lower limit of quantification (LLOQ) (0.500 ng/mL in fat homogenate, which is equivalent to <10.0 ng/g of fat) after the 13‐week treatment‐free period (CSR, NewAmsterdam Pharma).

### 3.2 Clinical trials

#### 3.2.1 Phase I clinical trials assessing obicetrapib PK parameters

**FIGURE 2:**
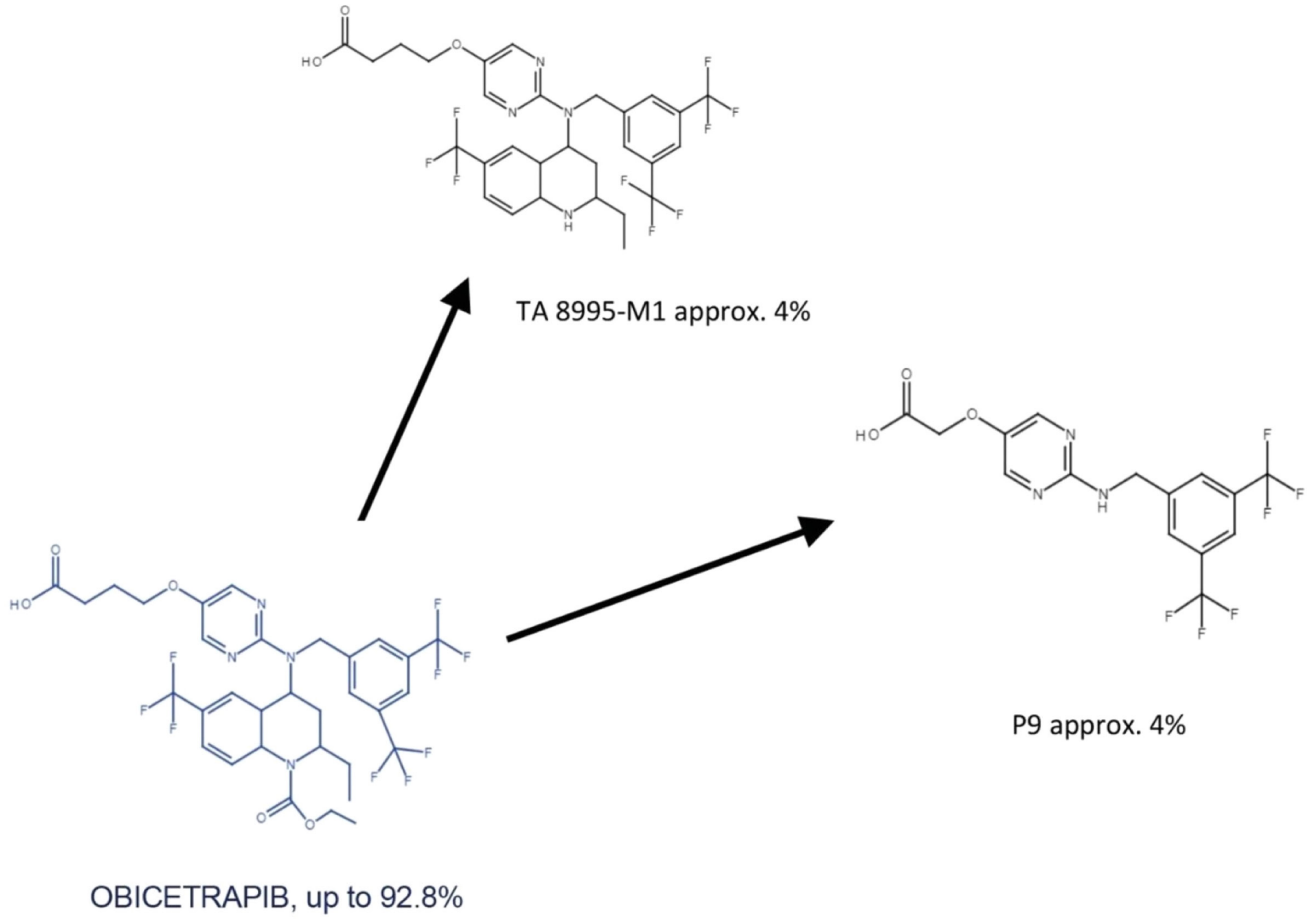
Obicetrapib (TA‐8995) metabolites detected above LLOQ (>1% radioactivity) in human plasma expressed as the percent radioactivity profiled^†^ (TA‐8995‐07 CSR, NewAmsterdam Pharma). ^†^Data from *n* = 6 individuals from 24 h AUC pools. AUC, area under the curve; LLOQ, lower limit of quantification; M, metabolite; P, secondary metabolite.

Figure 2 illustrates the obicetrapib (TA‐8995) M1 detected above the LLOQ (>1% radioactivity) in human plasma expressed as the percent radioactivity profiled. A study (NCT02408055) was conducted in healthy male participants (*n* = 6) to assess the absorption, metabolism, and excretion of a single dose of 10 mg of ^14^C‐obicetrapib (TA‐8995‐07 CSR, NewAmsterdam Pharma). One of the objectives of the study was to determine the chemical structure of ^14^C‐obicetrapib M1 that represent ≥10% of total radioactivity in plasma. The participants received 10 mL ^14^C‐obicetrapib oral suspension, containing 10 mg and 100 μCi of ^14^C‐obicetrapib in the fasted state. Clinical samples for analysis were selected based upon the levels of radioactivity present as determined by liquid scintillation counting and were stored at below −50°C (nominally −80°C) when not in use. Plasma samples from the participants were sequentially extracted with acetonitrile at five time points. The resulting extracts were concentrated by evaporation and reconstituted in mobile phase HPLC prior to analysis by radio‐HPLC. All samples were analyzed by liquid scintillation counting to evaluate the recovery of radioactivity. The profiles of the radiolabeled components in each sample of plasma were determined by HPLC analysis with off‐line radio‐detection. Selected samples from each matrix were then further analyzed by high‐resolution mass spectrometry to investigate the identity of M1. In plasma, the predominant metabolite component was unchanged obicetrapib (~79% of total drug‐related exposure), and the most abundant plasma M1 were M1 and P9 of which each accounted for ~4% of total exposure (Figure 2). All other peaks accounted for <3% of total exposure (not shown in Figure 2). No significant differences in the metabolite profiles were observed across the samples obtained from the six participants (TA‐8995‐07 CSR, NewAmsterdam Pharma).

Table 2 summarizes the end‐of‐treatment PK parameter results from the Phase I trials for the 5, 10, and 25 mg doses of obicetrapib for selected groups.^9^ The results for these groups are presented in the table because the 5 and 10 mg doses were used in subsequent Phase II trials and the 25 mg dose illustrates the dose‐dependent influence of obicetrapib. In the participants in groups 1–6 in the first trial (single ascending dose trial with 12 groups total), obicetrapib was absorbed and the time to maximum plasma concentration (*t*_max_) was achieved within 3 h for the 5 and 10 mg doses and 3.5 h for the 25 mg dose. The *t*_max_ for the 50–150 mg dosages was 4 h (data not shown). The *C*_max_ was 138, 251, and 467 ng/mL, and the AUC_(0‐24h)_ was 1816, 3580, and 5952 ng·h/mL for the 5, 10, and 25 mg doses of obicetrapib, respectively. There was moderate variability in plasma concentrations within each dose group, as indicated by the coefficients of variation that were 33%, 23%, and 30% for *C*_max_, and 34%, 21%, and 26% for AUC_(0‐24h)_ for the 5, 10, and 25 mg doses, respectively. There was similar variability in the *C*_max_ and AUC_(0‐24h)_ for the higher doses of obicetrapib, except the 100 mg dose, which had greater variability during the first 24 h after dosing (data not shown).^9^ The mean *t*_1/2_ was 125, 124, and 162 h for the 5, 10, and 25 mg doses, respectively (Table 2). Based on the PK assessment results of the other groups, there were no clinically relevant effects of food, age, gender, or ethnicity on the PK parameters of obicetrapib (data not shown). Obicetrapib was not detected in the urine of any participant.^9^

**TABLE 2:** Summary of end‐of‐treatment pharmacokinetic parameter results^a^ for obicetrapib (TA‐8995) from Phase I trials^9^ (TA‐8995‐09 CSR, NewAmsterdam Pharma).

Study	Treatment	*N*	*C*_max_ (ng/mL)	*t*_max_ (h)	*t*_½_ (h)	AUC _(0–24 h)_ (ng·h/mL)	R_ac_ ^b^
PK Assessment Trial							
Study 1—SAD^c^	Obicetrapib 5 mg	6	138 (33%)	3 (2, 3)	125 (22%)	1816 (34%)	‐
Study 1—SAD^c^	Obicetrapib 10 mg	6	251 (23%)	3 (2, 5)	124 (20%)	3580 (21%)	‐
Study 1—SAD^c^	Obicetrapib 25 mg	6	467 (30%)	3.5 (2, 6)	162 (19%)	5952 (26%)	‐
Study 2—SAD^d^	Obicetrapib 5 mg	10	116 (13%)	6 (4, 8)	‐	1796 (14%)	‐
Study 2—SAD^d^	Obicetrapib 10 mg	10	245 (14%)	6 (4, 8)	‐	3471 (18%)	‐
Study 2—SAD^d^	Obicetrapib 25 mg	10	750 (19%)	6 (3, 8)	‐	10 437 (21%)	‐
Study 2—MAD^e^	Obicetrapib 5 mg	10	436 (18%)	4 (3, 8)	148 (30%)	7606 (20%)^f^	4.3 (1.1)
Study 2—MAD^e^	Obicetrapib 10 mg	10	692 (23%)	6 (4, 8)	131 (19%)	12 707 (27%)^f^	3.7 (0.8)
Study 2—MAD^e^	Obicetrapib 25 mg	10	1398 (18%)	4 (2, 6)	121 (23%)	21 493 (21%)^f^	2.1 (0.3)

Abbreviations: ‐, value not reported; AUC, area under the curve; *C*_max_, maximum plasma concentration; CI, confidence interval; CV, coefficient of variation; MAD, multiple ascending dose; PK, pharmacokinetic; R_ac_, accumulation ratio; SAD, single ascending dose; *t*_½_, terminal half‐life; *t*_max_, time to maximum plasma concentration.

^a^ Values are geometric mean (CV%) for *C*_max_ and AUC parameters, median (minimum, maximum) for *t*_max_, and arithmetic mean (CV%) for *t*_½_.

^b^ Accumulation ratio (R_ac_) calculated as AUC_(0,τ,ss)_/AUC_(0–24 h)_, where τ is the dosing interval (24 h).

^c^ Study 1: Single oral doses in healthy White male participants under fasting conditions.

^d^ Study 2: Single oral doses in healthy White male participants after a standard breakfast.

^e^ Study 2 ‐ steady‐state assessment: Multiple doses in health White male participants after a standard breakfast; assessed after the last dose: day 42 for 5 mg obicetrapib and day 35 for other doses.

^f^ AUC (0, τ, ss) (ng ml^−1^ h), where τ is the dosing interval and ss is steady state.

In the second trial (single daily dose followed by repeated daily doses in five groups of healthy White males [*n* = 10]), plasma concentrations of obicetrapib increased approximately proportionally to dose with the single ascending doses and non‐proportionally in the multiple ascending doses (Table 2). The *t*_max_ of obicetrapib was 6 h with a single dose regardless of the strength of the dose (1–25 mg) and 4 h with multiple daily doses for all doses except 10 mg, which had a *t*_max_ of 6 h. The mean *t*_1/2_ of obicetrapib was 148, 131, and 121 h for the 5, 10, and 25 mg doses, respectively. With multiple once daily doses of obicetrapib, the R_ac_ indicated moderate accumulation (2 ≤ R_ac_ <5)^37^ in a dose‐dependent manner: 4.3 increase at 5 mg, 3.7 increase 10 mg, and 2.1 increase at 25 mg (Table 2). Visual inspection of trough concentrations suggested plasma levels of obicetrapib were approaching steady state within one to two weeks. Obicetrapib was not detected in the urine of any participant with multiple doses. In summary, the results from these PK analyses indicate that obicetrapib is absorbed, peak plasma concentrations typically occur within 4 to 6 h after oral dosing, and the mean *t*_1/2_ is between 121 and 148 h for 5, 10, and 25 mg doses.^9^

#### 3.2.2 Phase I trial assessing food effect on obicetrapib bioavailability (TA‐8995‐09)

Based on the plasma concentration data for obicetrapib, the peak and overall systemic exposure were increased ~1.6 fold under fed conditions compared to fasted conditions. The least‐squares geometric mean of fed versus fasted ratios were 154.9%, 155.4%, and 158.5% for AUC_0‐t_, AUC_0‐∞_, and *C*_max_, respectively (Table 3). The associated intra‐subject coefficients of variation were 34.6%, 36.8%, and 46.7% for AUC_0‐t_, AUC_0‐∞_, and *C*_max_, respectively. Based on the review of the individual participant data (not shown in table), 19 of 23 participants (~80%) had fed versus fasted ratios >125% for AUC. Similarly, 17 of 23 participants (~74%) had fed versus fasted ratios >125% for *C*_max_. The median (minimum, maximum) *t*_max_ of obicetrapib was 5.5 (2.5, 12) h and 5.0 (2.5, 48) h under fed and fasted conditions (data not shown in table) (TA‐8995‐09 CSR, NewAmsterdam Pharma).

**TABLE 3:** Summary of pharmacokinetic parameter results from a Phase I food effect trial using 10 mg obicetrapib (*n* = 23) (TA‐8995‐09 CSR, NewAmsterdam Pharma).

Parameters	Treatment	LS GM	LSGM Ratio (90% CI) (Fed vs. Fasted)	ISCV (%)
*C*_max_ (ng/mL)	Fed	222	159 (126, 199)	46.7
	Fasted	140		
AUC(_0‐t_) (ng·h/mL)	Fed	19 190	155 (130, 184)	34.6
	Fasted	12 390		
AUC(_0‐∞_) (ng·h/mL)	Fed	25 800	155 (129, 187)	36.8
	Fasted	16 600		

Abbreviations: AUC, area under the curve; *C*_max_, maximum plasma concentration; CI, confidence interval; ISCV, intra‐subject coefficients of variation; LSGM, least squares geometric means.

#### 3.2.3 Phase II clinical trials

Table 4 summarizes the end‐of‐treatment and final post‐treatment PK parameter results for the 2.5, 5, and 10 mg doses of obicetrapib in four Phase II trials.

**TABLE 4:** Summary of end‐of‐treatment and post‐treatment pharmacokinetic parameter results for obicetrapib for the pharmacokinetic populations of Phase II trials^10^, ^11^, ^12^, ^13^ (Data on file, NewAmsterdam Pharma).

Study and Treatment	PK Assessment Visit	N	Mean (SD) Plasma Level (ng/mL)	% Change in Plasma Level
TULIP				
Obicetrapib 5 mg	Week 12 (EOT)	39	257.6 (56.7)	
	Week 20^a^	40	5.9 (3.0)	−97.8 (0.8)
Obicetrapib 10 mg	Week 12 (EOT)	35	407.5 (86.5)	
	Week 20^a^	37	9.8 (4.2)	−97.7 (0.7)
Obicetrapib 10 mg + AT20	Week 12 (EOT)	38	404.0 (92.2)	
	Week 20^a^	38	10.8 (5.9)	−97.4 (1.0)
Obicetrapib 10 mg + RO10	Week 12 (EOT)	39	402.0 (132.7)	
	Week 20^a^	39	11.3 (7.4)	−97.3 (1.3)
ROSE				
Obicetrapib 5 mg + HIS^b^	Week 8 (EOT)	39	254.1 (104.0)	
	Week 23^c^	39	2.9 (2.0)	−98.5 (1.7)
Obicetrapib 10 mg + HIS^b^	Week 8 (EOT)	40	394.7 (130.9)	
	Week 23^c^	40	5.6 (4.3)	−98.4 (1.9)
ROSE2				
Obicetrapib 10 mg + HIS^b^	Week 12 (EOT)	26	387.4 (121.2)	
	Week 16^d^	25	24.5 (18.6)	−93.7 (3.3)
Obicetrapib 10 mg + ezetimibe 10 mg + HIS^b^	Week 12 (EOT)	31	360.4 (97.2)	
	Week 16^d^	31	28.7 (16.7)	−92.2 (3.7)
TA‐8995‐203				
Obicetrapib 2.5 mg + SST^e^	Week 8 (EOT)	25	194.5 (42.7)	
	Week 12^d^	25	13.2 (6.5)	−93.4 (2.6)
Obicetrapib 5 mg + SST^e^	Week 8 (EOT)	25	362.3 (118.2)	
	Week 12^d^	25	24.6 (14.9)	−93.5 (3.4)
Obicetrapib 10 mg + SST^e^	Week 8 (EOT)	26	506.3 (237.5)	
	Week 12^d^	26	27.6 (30.9)	−95.2 (2.3)

Abbreviations: AT20, atorvastatin 20 mg; EOT, end of treatment; HIS, high‐intensity statin therapy; PK, pharmacokinetic; RO10, rosuvastatin 10 mg; ROSE, Randomized Study of Obicetrapib as an Adjunct to Statin Therapy; ROSE2, Study to Evaluate the Effect of Obicetrapib in Combination with Ezetimibe as an Adjunct to High‐Intensity Statin Therapy; SD, standard deviation; SST, stable statin therapy; TA‐8995‐203, A Dose‐Finding Study in Japanese Patients to Evaluate the Effect of Obicetrapib as an Adjunct to Stable Statin Therapy; TULIP, TA‐8995: Its Use in Patients with Mild Dyslipidaemia.

^a^ Eight weeks after the end of treatment.

^b^ High‐intensity statin dose was atorvastatin 40 mg or 80 mg/day or rosuvastatin 20 mg or 40 mg/day.

^c^ Fifteen weeks after the end of treatment.

^d^ Four weeks after the end of treatment.

^e^ Stable statin therapy was atorvastatin 10 or 20 mg/day or rosuvastatin 5 or 10 mg/day for at least eight weeks prior to screening.

#### 3.2.4 TULIP

The mean (standard deviation [SD]) plasma levels of obicetrapib at Week 12 (end of treatment) were 257.6 (56.7) ng/mL for the 5 mg dose group and between 402.0 (132.7) and 407.5 (86.5) ng/mL for the 10 mg obicetrapib dose groups (monotherapy and in combination with atorvastatin or rosuvastatin), which indicated a dose‐dependent effect. Eight weeks after the last dose of obicetrapib (Week 20), mean (SD) plasma concentrations were 5.9 (3.0) ng/mL for the 5 mg dose group and between 9.8 (4.2) and 11.3 (7.4) ng/mL for the 10 mg dose groups, demonstrating that levels decreased by approximately 97% for all treatment groups (Table 4). Furthermore, obicetrapib was not detected in the urine of any of the participants (TA‐8995‐03 CSR, NewAmsterdam Pharma).

#### 3.2.5 ROSE

At Week 8 (end of treatment), the mean (SD) plasma level of obicetrapib was 254.1 (104.0) ng/mL for the 5 mg dose group and 394.7 (130.9) ng/mL for the 10 mg dose group. The plasma levels of obicetrapib decreased at each post‐end of treatment assessment with a mean (SD) plasma level of obicetrapib of 2.9 (2.0) ng/mL for the 5 mg dose group and 5.6 (4.3) ng/mL for the 10 mg dose group at Week 23 (15 weeks after the end of treatment). The mean percent change in the plasma level of obicetrapib from Week 8 to Week 23 was −98.5% for the 5 mg dose group and − 98.4% for the 10 mg dose group (Table 4), indicating almost complete systemic elimination 15 weeks after the end of treatment^11^ (TA‐8995‐201 CSR, NewAmsterdam Pharma).

#### 3.2.6 ROSE2

At Week 12 (end of treatment), the mean (SD) plasma level of obicetrapib was 387.4 (121.2) ng/mL for the 10 mg obicetrapib group and 360.4 (97.2) ng/mL for the 10 mg obicetrapib plus 10 mg ezetimibe group for the on‐treatment PK population. At Week 16 (4 weeks after the end of treatment), the mean (SD) plasma level of obicetrapib was 24.5 (18.6) ng/mL for the 10 mg obicetrapib group and 28.7 (16.7) ng/mL for the 10 mg obicetrapib plus 10 mg ezetimibe group. The mean percent change in plasma levels of obicetrapib from Week 12 to Week 16 (4 weeks after the end of treatment) was −93.7% for the 10 mg obicetrapib group and − 92.2% for the 10 mg obicetrapib plus 10 mg ezetimibe group (Table 4). These results demonstrate that very low levels of obicetrapib were present and indicate that it had been mostly eliminated 4 weeks after the end of treatment^12^ (TA‐8995‐202 CSR, NewAmsterdam Pharma).

#### 3.2.7 TA‐8995‐203

At Week 8 (end of treatment), the mean (SD) plasma level of obicetrapib was 194.5 (42.7) ng/mL for the 2.5 mg dose group, 362.3 (118.2) ng/mL for the 5 mg dose group, and 506.3 (237.5) ng/mL for the 10 mg dose group. At Week 12 (4 weeks after the end of treatment), the mean plasma level of obicetrapib was 13.2 (6.5) ng/mL for the 2.5 mg dose group, 24.6 (14.9) ng/mL for the 5 mg dose group, and 27.6 (30.9) ng/mL for the 10 mg dose group. The mean percent change in plasma levels of obicetrapib from Week 8 to Week 12 was −93.4%, −93.5%, and −95.2% for the 2.5 mg, 5 mg, and 10 mg dose groups, respectively (Table 4). Similar to findings from ROSE and ROSE2, these results demonstrate that very low levels of obicetrapib were present and indicate that it had been mostly eliminated 4 weeks after the end of treatment^13^ (TA‐8995‐203 CSR, NewAmsterdam Pharma).

## 4 DISCUSSION

Based on the results of the non‐human primate study and Phase I and Phase II clinical trials, obicetrapib is absorbed efficiently and does not exhibit prolonged systemic elimination.^9^, ^10^, ^11^, ^12^, ^13^ The results of the 9‐month dosing non‐human primate study showed complete systemic elimination of obicetrapib after a 13‐week treatment‐free period. Furthermore, after doses administered up to 50 mg/kg/day, obicetrapib was not detected in monkey adipose tissue after the 13‐week recovery period (CSR, NewAmsterdam Pharma), indicating that obicetrapib did not accumulate in adipose tissue in contrast to what was observed with longer‐term trials of anacetrapib. The results from Phase I and Phase II clinical trials demonstrated moderate accumulation during treatment periods of between three and 12 weeks and more rapid systemic elimination after the end of treatment compared to some other agents in the CETP inhibitor class, particularly anacetrapib. A 92–98% reduction in plasma levels of obicetrapib was achieved within four to 15 weeks after the end of treatment^9^ (TA‐8995‐03 CSR; TA‐8995‐201 CSR; TA‐8995‐202 CSR; TA‐8995‐203 CSR, NewAmsterdam Pharma).

The results of a Phase I single ascending dose trial that included an investigation of obicetrapib PK parameters during fed versus fasted conditions at a 50 mg dose in a subset of participants (*n* = 5 White healthy males) indicated that food did not have a clinically relevant effect on the AUC or *C*_max_ of obicetrapib.^9^ However, the results of a dedicated Phase I trial to evaluate the effect of food on the bioavailability of 10 mg obicetrapib demonstrated that the AUC_(0‐∞)_ and *C*_max_ were both increased 1.6‐fold when obicetrapib was taken with a high‐fat meal, compared to fasting conditions, in healthy adult males and females (*n* = 23) (TA‐8995‐09 CSR, NewAmsterdam Pharma). The food effect observed with obicetrapib does not appear to be clinically relevant based on results from the Phase II trials, which indicated similar plasma level changes whether obicetrapib was taken with food^10^, ^11^ or without food.^12^, ^13^ In comparison to obicetrapib, a high‐fat meal increased the AUC _(0‐∞)_ and *C*_max_ of anacetrapib by 6‐and 8‐fold, respectively, compared to a fasting state.^24^ A food effect was also observed with the other CETP inhibitors. Administration in a fed state affected torcetrapib absorption substantially, with its mean exposure increased by 20‐ to 30‐fold after a meal.^28^ The food effect for dalcetrapib and evacetrapib was similar to that observed with obicetrapib. A high‐fat meal increased the AUC_(0–36)_ and *C*_max_ of dalcetrapib by 1.6‐fold and 2.3‐fold, respectively, compared to fasting conditions,^29^ and a high‐fat meal increased the AUC_(τ)_ and *C*_max_ of evacetrapib by 1.4‐fold and 1.5‐fold, respectively, compared to fasting conditions.^30^

There are explanations for the longer *t*_1/2_ and accumulation in adipose tissue that have been observed with anacetrapib but not with other previous CETP inhibitors or obicetrapib. The *t*_1/2_ of anacetrapib was determined to be relatively short (up to 80 h) in initial Phase I trials that were of a short duration (2 weeks),^19^ whereas, in a trial with a treatment duration of 8 weeks, the *t*_1/2_ of anacetrapib was observed to be three to four weeks.^20^ Anacetrapib was detected in plasma up to 4 years after the last treatment dose in the 76‐week DEFINE trial.^21^ It has been hypothesized that the increased half‐life observed for anacetrapib with longer versus shorter treatment durations is related to sampling and analytical limitations and not to actual differences in PK. Longer treatment durations likely improve the ability to identify the true terminal phase of a study drug.^22^ The half‐life of a drug is also related to its clearance and distribution volume, with a long half‐life resulting from low clearance and/or a large volume of distribution.^22^ Anacetrapib accumulates in adipose tissue, and its concentration exhibits a slow decline, resulting in a large reservoir of the drug.^17^ Thus, the distribution to and the slow elimination from adipose tissue is the primary explanation for the observed extended terminal elimination half‐life of anacetrapib.^17^, ^22^

**FIGURE 3:**
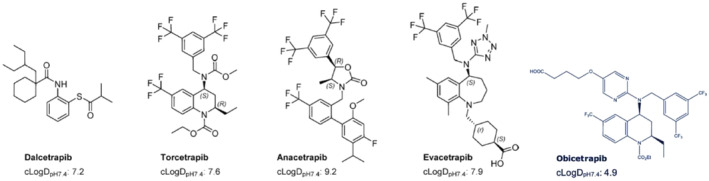
Chemical structures and lipophilicity of CETP inhibitors.^7^, ^17^, ^38^, ^54^

The accumulation in adipose tissue is unique to anacetrapib. The potential mechanisms that might explain why anacetrapib accumulated in adipose tissue are related to its physicochemical properties. Anacetrapib is the most lipophilic of the previous CETP inhibitors, with a cLogD_pH7.4_ of 9.2 compared to a cLogD_pH7.4_ of 7.2, 7.6, and 7.9 for dalcetrapib, torcetrapib, and evacetrapib respectively (Figure 3).^7^, ^38^ Additionally, anacetrapib has the lowest PSA of the previous CETP inhibitors, with a PSA of 39 Å^2^ compared to 46, 59, and 87 Å^2^ for dalcetrapib, torcetrapib, and evacetrapib, respectively.^23^ Therefore, the accumulation of anacetrapib in adipose tissue is most likely due to its high lipophilicity and poor aqueous solubility.^17^, ^22^ These physicochemical properties can also explain the large food effect that was observed with anacetrapib.^22^, ^23^

In contrast to the physicochemical properties of anacetrapib, as a tetrahydroquinoline derivative, obicetrapib is less lipophilic (exhibits a cLogD_pH7.4_ of 4.9)^7^ and has a PSA of 105 Å^2^,^39^ which might explain the differences in *t*_1/2_ and accumulation seen between the two CETP inhibitors. Whereas an 8‐week treatment duration of anacetrapib indicated it had a *t*_1/2_ of three to four weeks,^20^ the results of a Phase 1 trial of obicetrapib with a 3‐ to 4‐week treatment duration at doses of 1–25 mg in healthy participants demonstrated a *t*_1/2_ between 121 and 151 h.^9^ The physicochemical properties of obicetrapib support the results of the PK assessments, which demonstrate it has little accumulation without any clinical relevance and no delayed elimination from the systemic circulation after the last treatment dose. The on‐going Phase III trials of obicetrapib that have longer treatment durations (1 to 2.5 years) will allow further investigation of its elimination.

A single dosage of 10 mg/day of obicetrapib has been selected for later phase development based on the PK and pharmacodynamic properties observed at that dosage in the Phase I and Phase II trials. The totality of the evidence to date for obicetrapib supports chronic dosing up to 10 mg once daily and continued clinical development for dyslipidemia management and preventing atherosclerotic cardiovascular disease (ASCVD). Although statins are the first‐line pharmacotherapy for the management of elevated LDL‐C levels and several nonstatin therapies are available to address residual ASCVD risk, many patients may not achieve their LDL‐C therapeutic objectives, especially very high‐risk patients for whom the recommended LDL‐C level is <55 mg/dL.^40^, ^41^, ^42^ The results of analyses that examined the use of lipid‐lowering therapies in the GOULD and DA VINCI cohorts showed that only 10–22% of high‐ and very‐ high‐risk patients achieve their LDL‐C therapeutic objective, leaving ~80–90% of patients at high‐risk for a cardiovascular event with inadequate treatment of LDL‐C.^43^, ^44^, ^45^, ^46^ The results of other observational studies have indicated that patients with an LDL‐C therapeutic objective <70 mg/dL are not able to achieve that threshold with a statin or statin plus ezetimibe combination.^47^ Furthermore, analyses from modeling studies suggest that most patients who require intensive LDL‐C lowering to achieve LDL‐C levels <55 mg/dL will require pharmacotherapy beyond a statin plus ezetimibe combination.^46^, ^48^

With its ability to significantly reduce ApoB‐containing lipoprotein levels and a safety profile similar to placebo, obicetrapib has the potential to be a new agent in the armamentarium for dyslipidemia management and ASCVD risk reduction. Four Phase III trials of obicetrapib are currently in progress: the Randomized Study to Evaluate the Effect of Obicetrapib on Top of Maximum Tolerated Lipid‐Modifying Therapies (BROADWAY, NCT05142722),^49^, ^50^ the Evaluate the Effect of Obicetrapib in Patients with Heterozygous Familial Hypercholesterolemia on Top of Maximum Tolerated Lipid‐Modifying Therapies (BROOKLYN, NCT05425745) trial,^50^, ^51^ the Study of Obicetrapib and Ezetimibe Fixed Dose Combination on Top of Maximum Tolerated Lipid‐Modifying Therapies (TANDEM, NCT06005597),^52^ and the Cardiovascular Outcome Study to Evaluate the Effect of Obicetrapib in Patients with Cardiovascular Disease (PREVAIL, NCT05202509).^53^ The results of these trials will contribute to the evidence of the efficacy and safety of obicetrapib and further support its clinical development as the first‐in‐class CETP inhibitor available for clinical use.

In conclusion, the results of toxicokinetic assessments from a pre‐clinical experiment in nonhuman primates demonstrate complete, or near complete, systemic elimination and no accumulation of obicetrapib in adipose tissue 13 weeks after the last treatment dose. Similarly, the results of clinical trials in humans demonstrate that obicetrapib shows modest accumulation without clinical relevance and is nearly completely eliminated from the systemic circulation 4 weeks after the last treatment dose. Plasma levels of obicetrapib increase with food intake to levels comparable with dalcetrapib and evacetrapib, and with a much smaller increase than observed with torcetrapib and anacetrapib. The results of these studies provide further evidence supporting the safety of obicetrapib and support the once‐daily, chronic dosing of 10 mg in patients requiring further LDL‐C lowering to achieve their therapeutic objectives.

## AUTHOR CONTRIBUTIONS

*Participated in research design*: J. Kastelein, M. Davidson, M. Ditmarsch, A. Hsieh. *Conducted experiments*: S. Nicholls, A. Nelson. *Wrote or contributed to the writing of the manuscript*: S. Nicholls, A. Nelson, J. Kastelein, M. Ditmarsch, A. Hsieh, J. Johnson, D. Curcio, D. Kling, C. Kirkpatrick, M. Davidson. All authors approved the final version of the manuscript.

## CONFLICT OF INTEREST STATEMENT

**Stephen J. Nicholls** received grant/research support from AstraZeneca, NewAmsterdam Pharma, Amgen, Anthera, Eli Lilly, Esperion, Novartis, Cerenis, The Medicines Company, Resverlogix, InfraReDx, Roche, Sanofi‐Regeneron, and LipoScience; and was a consultant for AstraZeneca, Amarin, Akcea, Eli Lilly, Anthera, Omthera, Merck, Takeda, Resverlogix, Sanofi‐Regeneron, CSL Behring, Esperion, Boehringer Ingelheim, and Vaxxinity. **Adam J. Nelson** received personal fees from Boehringer Ingelheim, AstraZeneca, Amgen, Novartis, and Sanofi. **John J.P. Kastelein** is Chief Scientific Officer for NewAmsterdam Pharma and Emeritus Professor of Medicine at the University of Amsterdam, The Netherlands. **Marc Ditmarsch** is Chief Development Officer for NewAmsterdam Pharma. **Andrew Hsieh** is Vice President of Medical Affairs for NewAmsterdam Pharma. **Judith Johnson** is Executive Director of Clinical Operations for NewAmsterdam Pharma. **Danielle Curcio** is Executive Director of Clinical Operations for NewAmsterdam Pharma. **Douglas Kling** is Chief Operating Officer for NewAmsterdam Pharma. **Carol F. Kirkpatrick** is an employee of Midwest Biomedical Research, which has received consulting fees and/or grant funding from Acasti Pharma, Beren Therapeutics, Eli Lilly and Company, Indiana University and Foundation, Matinas BioPharma, NewAmsterdam Pharma, NorthSea Therapeutics, and 89Bio. **Michael H. Davidson** is Chief Executive Officer for NewAmsterdam Pharma.

## ETHICS STATEMENT

The pre‐clinical trials were conducted in accordance with the Good Laboratory Practice Regulations 1999 and the requirements of the Animals (Scientific Procedures) Act 1986, and local ethical reviews were maintained. The clinical trials were performed in accordance with the Declaration of Helsinki, Good Clinical Practice Guidelines, and applicable regulatory requirements. Approval was obtained from the relevant ethical review boards for each research site.

## Data Availability

The authors declare that the data supporting the findings of the studies discussed in this article are contained within the article, except when indicated with, “data not shown.” Selected data were discussed in the text but not included in tables to maintain brevity. Other selected data are not included in this article because they are available in previous publications. NewAmsterdam Pharma is committed to sharing, with qualified external researchers, access to individual‐level data and supporting clinical documents from eligible studies. The data that support the findings of this study are available from the corresponding author upon reasonable request. These requests are reviewed and approved by an independent review panel on the basis of scientific merit. All data provided are anonymized, in line with applicable laws and regulations.
